# Fever Hospitals and the Profession

**Published:** 1897-08-07

**Authors:** 


Vol. XXII.—No. 567.] [Aug. 7, 1897.

### Fever Hospitals and the Profession.

The steadily increasing demand for admission to the hospitals under the management of the Metropolitan Asylums Board is a matter of considerable importance to the medical profession at large, as well as to the ratepayer. Ten years ago the Board treated 2,000 cases, all of which were classed as paupers, while last year 22,000 cases were admitted, collected from various classes of society. Year by year new hospitals are being built, but the more there are the greater seems to be the number of the patients who are anxious for admission to them, and the greater the assumed necessity for continuing to build.

To the ratepayer this is becoming an anxious question. All this extension of hospital building has taken place in the absence of any great epidemic of disease. Meanwhile the public are being trained to look upon hospital treatment as their right, and if an epidemic should arise it is not difficult to foresee that the provision of accommodation for all who demand admission would become an intolerable burden. To the medical profession the sweeping of all these fever cases into great hospitals already means a very serious annual loss, a loss the magnitude of which in the future it is difficult even to guess at. What we have to recognise is that by the hospital system it is possible for five hundred patients at a time to be treated by, say four, medical men at comparatively small salaries, instead of being distributed among, and bringing fees to, scores of practitioners outside. This may be thought by some to be an ignoble way of looking at the matter, but it is an aspect of the question which is forcing itself more and more on those who have to live by general practice. What the general practitioner finds is that all the emergency work falls upon him as before. The sudden call to see the urgent case, the anxious responsibility of deciding on its nature, the odium which attaches to its notification, all these still are his as in days before the hospitals. In those days, however, the doctor did get some credit and some monetary reward for his work. He had to attend throughout the illness, and such honour and such fees as the cure of his patient might produce at least were his. Now, however, no sooner is the emergency over than the patient is spirited off to a hospital, where he is treated on co-operative principles. Medical men quite as much as ratepayers may well ask where is all this going to end. What is clear is that the end has not come yet, and that many stripes will yet be laid upon our unfortunate profession, unless the ratepayers should perchance kick against the hardly concealed intention of the Asylums Board to draw to itself the treatment of the whole of the fever of London. What is happening is that year by year people of a higher social status are accepting the undoubted convenience offered to them by the Asylums Board hospitals, and we do not think it will be very long before a claim is put forward for the provision of a higher class of accommodation, or rather of a more select companionship for these better-class patients. This is no new question. Hospital managers have again and again felt the hardship of placing patients, who have been delicately and nicely brought up, next to others who may have come from the lowest slums. By the exercise of care and judgment this hardship has been lessened as far as possible, but it is obvious to every one that it exists. People hear of the good management of the fever hospitals, and they send their children to them without thought of their possible surroundings. Surely it does not occur to them that, but for the care and watchfulness of the medical officers and the nurses in charge, an innocent and well brought up girl may be placed in the next bed to a foul-mouthed prostitute who may sputter blasphemies and obscenities through the days and nights of her delirium.

We do not think we are wrong in saying that there has for some time been a desire among the officials for greater power of classification. Everyone who knows about the treatment of fevers knows how much depends upon feeding, and also knows how appetite among the nicely brought up depends upon nice surroundings; and any medical officer who should report that the recovery of some of his patients was retarded by their being forced to mix with others of a very different social grade would certainly only be reporting the exact truth. Classification, then, is likely to be asked for as a medical necessity so soon as the managers overtake the present pressure on their beds; and so soon as classification become a fact, and patients become able to engage private rooms in these hospitals, or even to secure admission to select wards, then another great social stratum will be swept into the hospital net, and, so far as their fevers are concerned, will be lost to the profession. We repeat then that we are not yet at the end, nor near it.

